# Fire ant social chromosomes: Differences in number, sequence and expression of odorant binding proteins

**DOI:** 10.1002/evl3.22

**Published:** 2017-08-23

**Authors:** Rodrigo Pracana, Ilya Levantis, Carlos Martínez‐Ruiz, Eckart Stolle, Anurag Priyam, Yannick Wurm

**Affiliations:** ^1^ School of Biological and Chemical Sciences Queen Mary University of London E1 4NS London United Kingdom

**Keywords:** Green beard, olfaction, pheromone, social behavior, social chromosome, *Solenopsis invicta*, supergene

## Abstract

Variation in social behavior is common yet our knowledge of the mechanisms underpinning its evolution is limited. The fire ant *Solenopsis invicta* provides a textbook example of a Mendelian element controlling social organization: alternate alleles of a genetic element first identified as encoding an odorant binding protein (OBP) named *Gp‐9* determine whether a colony accepts one or multiple queens. The potential roles of such a protein in perceiving olfactory cues and evidence of positive selection on its amino acid sequence made it an appealing candidate gene. However, we recently showed that recombination is suppressed between *Gp‐9* and hundreds of other genes as part of a >19 Mb supergene‐like region carried by a pair of social chromosomes. This finding raises the need to reassess the potential role of *Gp‐9*. We identify 23 OBPs in the fire ant genome assembly, including nine located in the region of suppressed recombination with *Gp‐9*. For six of these, the alleles carried by the two variants of the supergene‐like region differ in protein‐coding sequence and thus likely in function, with *Gp‐9* showing the strongest evidence of positive selection. We identify an additional OBP specific to the Sb variant of the region. Finally, we find that 14 OBPs are differentially expressed between single‐ and multiple‐queen colonies. These results are consistent with multiple OBPs playing a role in determining social structure.

**Impact Summary**

The invasive red fire ant provides a unique opportunity to investigate how changes in social behavior can evolve. In this species, two distinct forms of social organization coexist: colonies either have strictly one queen or up to dozens of reproductive queens.

Pioneering work in the early 2000s demonstrated that this social dimorphism has a genetic basis: one of the two alleles (versions) of the gene *Gp‐9* is always and exclusively present in multiple‐queen colonies but never in single‐queen colonies. *Gp‐9* encodes an odorant binding protein (OBP), a type of protein that can be involved in the production and in the perception of pheromones. The two alleles of *Gp‐9* differ in amino acid sequence, and thus likely also in function. Furthermore, workers with the *b* allele of *Gp‐9* behave spitefully toward queens that lack the *b* allele—in this sense the *b* allele appears to be a selfish gene with a so‐called “green beard” effect.

However, it was recently shown that the two alleles of *Gp‐9* are part of a pair of “social chromosomes.” Like a pair of sex chromosomes, the two social chromosomes differ from each other, so that hundreds of genes in addition to *Gp‐9* have alleles present exclusively in multiple‐queen colonies. This challenges the idea that *Gp‐9* is pivotal in determining social organization.

Here, to determine whether there is still reason to believe that OBPs play roles in this system, we characterize all fire ant OBPs. We find that fire ants have 24 OBPs, 10 of which are in the social chromosome. Two of the social chromosome OBPs are exclusive to alternate social chromosome variants, and four OBPs in addition to *Gp‐9* have differences in amino acid sequence between the variants. We also find differences in the activity levels of 14 OBP genes between single‐ and multiple‐queen colonies.

In sum, our study provides evidence that multiple OBPs may be responsible for differences between single‐ and multiple‐queen colonies. These results represent a significant step toward understanding the mechanisms by which the social chromosomes function.

## Introduction

Variation in social behavior is common yet our knowledge of the mechanisms underpinning its evolution is limited (Robinson et al. 2005; Johnson and Linksvayer 2010). The fire ant *Solenopsis invicta* provides a rare, textbook example of variation in a fundamental social trait: some colonies have one queen, whereas others have up to dozens of queens. Queens that will form their own single‐queen colony typically disperse over greater distances and can effectively colonize newly available habitats. In contrast, multiple‐queen colonies can outcompete single‐queen colonies in saturated habitats and harsh environments, and can split by fission (Bourke and Heinze 1994; Ross and Keller 1995; Tschinkel 2006). Multiple additional traits differ between the two social forms, including in queen fecundity, colony size, worker size distribution, and worker aggressiveness (Ross and Keller 1995; DeHeer et al. 1999; Keller and Ross 1999; Goodisman et al. 2000; DeHeer 2002; Buechel et al. 2014; Huang et al. 2014).

A series of landmark studies (Ross 1997; Keller and Ross 1998; Ross and Keller 1998) demonstrated that the two social forms are under the control of a Mendelian element. This element was first identified in a screen of electrophoretic markers as a polymorphic protein coding gene, *Gp‐9*, with two alleles: *Gp‐9B* and *Gp‐9b* (Ross 1997). If a colony includes only *Gp‐9 BB* workers, they will accept a single *Gp‐9 BB* queen and execute any additional queens. In contrast, if more than ∼20% of the workers in a colony are *Gp‐9 Bb* heterozygotes, they will execute reproductively active *Gp‐9 BB* queens but accept dozens of *Gp‐9 Bb* queens (Ross 1997; Keller and Ross 1998, 1999; Ross and Keller 1998, 2002; DeHeer et al. 1999; Gotzek & Ross 2007). In contrast, *Gp‐9 bb* queens die before becoming reproductively active (Ross 1997; DeHeer et al. 1999; Keller and Ross 1999; Gotzek and Ross 2007; Trible and Ross 2016). The workers discriminate between queens of alternate genotypes based on olfactory cues (Keller and Ross 1998; Ross and Keller 1998, 2002), such as differences in the queens’ cuticular hydrocarbon profiles (Eliyahu et al. 2011; Trible and Ross 2016). Because workers carrying the *Gp‐9b* allele recognize whether queens also carry this allele and execute those that do not, this system represents a rare example of a “green beard gene” (Keller and Ross 1998), named after a theoretical model of a behavioral selfish genetic element (West and Gardner 2010).

In another landmark study, Krieger and Ross (2002) demonstrated that *Gp‐9* encodes an odorant binding protein (OBP). OBPs are essential components of insect communication systems: they bind and transport pheromones and other semiochemicals, generally mediating their perception and sometimes their secretion (Pelosi et al. 2006, 2014; Leal 2013). Furthermore, tests of historical selection on *Gp‐9* reveal a significant excess of nonsynonymous (amino acid replacing) substitutions relative to synonymous (silent) substitutions between the lineage of *Gp‐9* b‐like alleles and *Gp‐9* B‐like alleles in the fire ant and its relatives. This implies that directional or diversifying selection has driven the molecular evolution of *Gp‐9*, and is associated with differentiation between the two forms of social organization in these ants (Krieger and Ross 2002, 2005). Several models lay out the potential function of *Gp‐9*, generally involving differential production or perception of pheromones in queens as well as workers of alternate genotypes (Krieger 2004; Gotzek and Ross 2007, 2009).

However, recent genome‐wide analyses of the social dimorphism revealed that the association between genotype and form of social organization is not limited to *Gp‐9* (Wang et al. 2013). Instead, genetic maps obtained using Restriction site Associated DNA (RAD) markers from crosses in seven families showed that this association extends over a large chromosomal region of suppressed recombination. The two variants of this region, respectively, marked by the *Gp‐9B* and *Gp‐9b* alleles are carried by a pair of “social chromosomes” named SB and Sb. The region is genetically differentiated over 10.8 Mb (55%) of the mapped assembly of the social chromosomes, although its total length could be 19.4–31.5 Mb given the estimated size of the nonassembled portion of the genome (Pracana et al. 2017). Based on the current NCBI gene set, this region contains at least 443 protein coding genes, including *Gp‐9*. The two chromosomes differ by at least one large inversion affecting a large portion of the region and an additional small (48 kb) inversion. The region of suppressed recombination can be described as a supergene, a locus containing multiple genes with tightly linked allelic combinations that control a complex polymorphic phenotype (Linksvayer et al. 2013; Schwander et al. 2014; Thompson and Jiggins 2014).

A study of general patterns of divergence and diversity showed that Sb has two orders of magnitude lower diversity than SB and than the rest of the genome, and that there is high ratio of nonsynonymous to synonymous substitutions between SB and Sb (Pracana et al. 2017). These results suggest that the evolution of Sb has been shaped by Hill–Robertson effects (the effects of selection on linked loci) due to the rarity of recombination in Sb (Wang et al. 2013; Pracana et al. 2017). However, little work has been done to characterize the genes present in the supergene region and to identify the mechanisms by which SB and Sb control the phenotypic differences between single‐ and multiple‐queen colonies. Studies using cDNA microarrays representing 3673 genes demonstrated that the supergene region is enriched for genes that are differentially expressed between queens (Nipitwattanaphon et al. 2014) and workers (Wang et al. 2008, 2013) of the two colony types. This suggests that genes other than *Gp‐9* could be responsible for the social dimorphism. Given that the determination of queen number requires the differential production and perception of semiochemicals by individuals of each genotype, it remains likely that OBPs play a part in determining the dimorphism.

Here, we determine to which extent OBPs have potentially functional divergence between social forms. For this, we identify all OBPs in the fire ant reference genome and map them to their genomic locations. Subsequently, we use population‐sequencing data to identify allelic differences between OBPs found on alternate variants of the social chromosome supergene. We also sequence an outgroup species, *Solenopsis geminata*, which allows us to determine which supergene variant carries the derived allele for each substitution. Finally, we compare gene expression profiles of all OBPs and gene coexpression modules between social forms. We show that there are nucleotide and amino acid sequence level differences between SB and Sb in the supergene OBPs, and that OBPs inside and outside the supergene are differentially expressed between single‐ and multiple‐queen colonies.

## Methods

### OBP DISCOVERY AND MANUAL GENE MODEL CURATION

The sequences of 18 fire ant OBP genes were previously reported, based on searches of Sanger‐sequenced Expressed Sequence Tag (EST) libraries (Table S1; Wang et al. 2007; Xu et al. 2009; Gotzek et al. 2011; Wurm et al. 2011). We used a curation approach similar to those previously used on other genes (Ingram et al. 2012; Corona et al. 2013; Kulmuni et al. 2013; Privman et al. 2013) to find the position of these OBP genes in the fire ant genome assembly (Wurm et al. 2011) and to discover previously unreported OBP genes. Our curation pipeline is described in detail in Supporting Information Methods. Briefly, we iteratively performed blastn and blastp (Camacho et al. 2009; Priyam et al. 2015) searches of the fire ant genome assembly (Wurm et al. 2011) using as queries the previously known fire ant OBP sequences as well as UniProt sequences that are part of the Pfam family “PBP_GOBP” (Finn et al. 2014; UniProt Consortium 2015). We manually curated the results of these searches by inspecting alignments of transcriptomic and genomic reads, which allowed us to infer intron–exon boundaries and coding sequences of these OBPs. We labeled the curated gene predictions that correspond to the previously known OBP genes (*SiOBP1–17*) according to the notation used by Gotzek et al. (2011) and we labeled newly discovered loci *SiOBPZ1–Z7*. We used a genetic map (Pracana et al. 2017) to assign OBPs to linkage groups. We generated a codon‐level alignment of the *S. invicta* OBPs using MAFFT‐linsi (version 6.903b; Katoh and Toh 2008) and PRANK (version 120626; Löytynoja and Goldman 2005), and built a phylogenetic tree using RaxML (version 8.2.9; Stamatakis 2006).

### IDENTIFYING ALLELIC DIFFERENCES FOR OBPs CARRIED BY ALTERNATE VARIANTS OF THE SOCIAL CHROMOSOME

We used whole‐genome sequences from one *SB* and one *Sb* male from each of seven colonies that had been sequenced at low coverage (Illumina 2*100 bp paired‐end genome shotgun sequences; ∼6×–8× coverage) in 2012 (NCBI SRP017317; Wang et al. 2013). Each of these samples is a haploid male (ants have a haplodiploid sex determination system). We filtered the reads, aligned them to the reference genome using bowtie2 (version 2.1.0; Langmead and Salzberg 2012), and used samtools and bcftools (version 1.3.1 for both; Li et al. 2009) to call variants among the individuals (Supporting Information Methods).

We produced whole‐genome sequencing reads of the outgroup species *S. geminata*. We sequenced a pool of 10 workers (sampled in Thailand by Dr. Adam Devenish, University College London, United Kingdom) using Illumina HiSeq 4000 (×11 coverage; Supporting Information Methods). We called variants between the sample and the reference assembly (using freebayes version 1.0.2‐33‐gdbb6160; Garrison and Marth 2012) within the coding sequence of each OBP using freebayes (Supporting Information Methods). We classed the alleles in each SB‐Sb substitution as ancestral or derived based on the allele carried in the outgroup species. We estimated the rate of synonymous and nonsynonymous divergence (dS and dN, respectively) between SB and Sb using seqinR (version 3.0‐7; Charif and Lobry 2007).

### DETECTION OF COPY NUMBER AND STRUCTURAL VARIATION IN OBPs

We visually inspected the alignments of the seven *SB* and the seven *Sb* haploid male samples against each OBP region. Deletions were identified as regions with no coverage and duplications were identified as regions where the coverage was higher than the background (Supporting Information Methods). Using the *de novo* assembler MIRA (version 4.0.2; Chevreux et al. 1999), we produced the sequence of the duplicate copy of *SiOBP12*, which we named *SiOBPZ5* (approach detailed in Supporting Information Methods).

### GENE EXPRESSION OF *S. INVICTA* OBPs IN PUBLICALLY AVAILABLE RNA SEQUENCING DATASETS

We analyzed all available RNA sequencing (RNA‐Seq) data from the NCBI SRA database for *S. invicta* (data from Wurm et al. 2011; Morandin et al. 2016 and PRJNA266847; details in Table S2). We determined the expression levels of *S. invicta* transcripts using the Kallisto count mode (version 0.43.0; Bray et al. 2016). Each sample was independently normalized using the DESeq2 method (version 1.14.1; Love et al. 2014). Additionally, we performed genome‐wide analysis of differential expression of data from Morandin et al. (2016), comparing three pools of queens from multiple‐queen colonies with two pools from single‐queen colonies, as well as two pools of workers from multiple‐queen colonies with three pools from single‐queen colonies. The pools of workers from multiple‐queen colonies contain a mix of individuals of both genotypes, whereas the pool of queens from multiple‐queen colonies has only *SB/SB* queens. We used a standard DESeq2 approach to identify expression differences between single‐ and multiple‐queen samples in queens and in workers. Additional details regarding these analyses are in Supporting Information Methods.

### DIFFERENTIAL EXPRESSION OF GENE COEXPRESSION MODULES ACROSS SOCIAL FORMS

We created gene coexpression modules from two cDNA microarray datasets (Platform GPL6930, with 25,344 probes representing 3673 genes; Supporting Information Methods and Table S3; Wang et al. 2007), one with queen samples (GSE42062; Nipitwattanaphon et al. 2013), the other with worker samples (E‐GEOD‐11694; Wang et al. 2008). Both datasets included *SB/SB* and *SB/Sb* samples. We created modules for each set using weighted gene coexpression network analysis (WGCNA) (version 1.49; Langfelder and Horvath 2008). We used *t*‐tests to determine whether any module eigengene is correlated with genotype or social form. In queens, we compared *SB/SB* to *SB/Sb* samples because all samples originate in multiple‐queen colonies. In workers, we separated the effect of genotype from the effect of social form following the approach in Wang et al. (2008): we compared genotypes (*SB/SB* vs *SB/Sb*) using samples from multiple‐queen colonies, and we compared across social forms (single queen vs multiple queen) using *SB/SB* samples only.

### EVIDENCE FOR SELECTION BASED ON NUCLEOTIDE DIVERSITY

Genomic regions that underwent recent selective sweeps are characterized by low nucleotide diversity (π) (Smith and Haigh 1974; Nei 1987; Nachman 2001). We used measurements of π along a sliding window of the genome, originally produced by Pracana et al. (2017), to identify selection pressure acting on *S. invicta* OBPs. Measurements of π were taken from nonoverlapping 10 kb windows (Supporting Information Methods).

## Results

### THE FIRE ANT REFERENCE GENOME ASSEMBLY CONTAINS 23 PUTATIVE OBPs

We combined automatic and manual curation approaches incorporating genomic and gene expression data to identify the sequence, exon structure, and location of 23 putative OBP genes in the *S. invicta* reference genome. Seventeen of these matched fire ant OBP gene sequences that had been previously reported, although with differences in sequence or in their inferred location in linkage groups (Table S1 and Supporting Information Methods). The remaining seven putative OBP genes are novel to *S. invicta* (Table S4). Interestingly, the coverage depth of *SiOBPZ6* is fourfold higher (95% confidence interval [3.66–4.78]; *t*‐test *t*_df = 6_ = 14.0, *P* < 10^−5^) than that of 1000 randomly selected genes, suggesting that there are four copies of this gene. There is little genetic variation among reads mapping to this gene across the 14 individuals in our dataset (4.2 Single Nucleotide Polymorphisms [SNPs] per 1000 bp). The alignment of whole‐genome sequencing reads of the outgroup species *S. geminata* to the *S. invicta* reference assembly shows that all OBPs are covered in this outgroup species. The coverage depth of *SiOBPZ6* is threefold higher in *S. geminata* (95% confidence interval [2.78–3.16]; *t*‐test *t*_df = 999_ = 20.7, *P* < 10^−15^), suggesting that this species also carries multiple copies of this gene.

**Figure 1:**
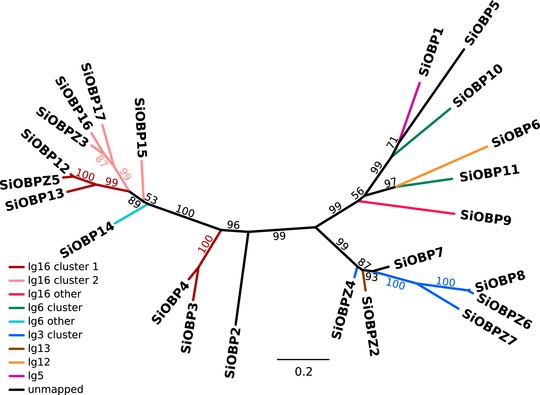
Phylogenetic tree based on a codon‐level alignment of revised gene predictions for previously described OBPs (*SiOBP1–17*) and novel OBPs (*SiOBPZ2–Z6*). Branches are colored by gene cluster and linkage group (lg). *SiOBPZ1* was removed from this analysis because the high divergence of its sequence led to unreliable alignments and positioning in the phylogeny. All OBPs on linkage group 16 (lg16) are within the supergene‐like region of the social chromosomes (Fig. 2).

Nine of the 23 OBPs in the genome are adjacent to unrelated genes, the remainder are organized into gene clusters. There are three locations in the genome each containing a cluster of four OBPs (two in linkage group 16, one in linkage group 3) and one containing a cluster of two OBPs (in linkage group 6). Intriguingly, none of these clusters appear to be completely monophyletic (Fig. 1). For previously known OBPs, the topology of our phylogenetic tree agrees with previously published trees (Gotzek et al. 2011; Zhang et al. 2016), with the exception of the position of *SiOBP15* (low bootstrap values in all trees) and *SiOBP5*.

### NONSYNONYMOUS DIFFERENTIATION BETWEEN SB And Sb IN OBPs

**Figure 2:**
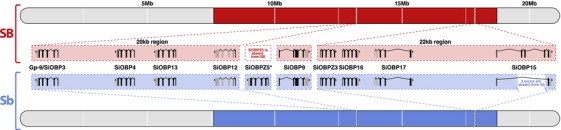
Relative positions on the social chromosome (i.e., linkage group 16) of 10 OBP loci, highlighting intron–exon structures and differences between the supergene region of Sb (blue) and SB (red). *SiOBPZ5* is specific to Sb but we do not know its exact location; *SiOBP15* is missing a 3‐exon region in Sb; *SiOBP9* is in an unmapped scaffold that likely belongs to the supergene region based on high levels of SB‐Sb differentiation (Pracana et al. 2017).

Eight of the OBPs are located in scaffolds of the SB fire ant genome assembly that map to the supergene region, with two clusters of four OBPs (Fig. 2). One of the clusters includes *Gp‐9* (which was named *SiOBP3* in Gotzek et al. 2011). A ninth gene, *SiOBP9*, is located in an unmapped scaffold that likely also belongs to the supergene region based on high levels of SB‐Sb differentiation (Fig. 2). To determine whether the supergene OBPs have allelic differences between SB and Sb, we used whole‐genome sequence data from seven *SB* males and seven *Sb* males.

These data confirmed the previous finding that *Gp‐9*/*SiOBP3* has eight nonsynonymous and one synonymous fixed single nucleotide substitutions between SB and Sb in the North American study population (Krieger and Ross 2002). Of the other OBPs in the supergene region, *SiOBP4* has three nonsynonymous and two synonymous substitutions. Two additional supergene OBPs have one fixed nonsynonymous substitution between SB and Sb (Table 1). Performing an analysis of the ratio of nonsynonymous to synonymous substitutions between alleles (dN/dS) was only possible for the two genes with the most divergent alleles: *Gp‐9*/*SiOBP3* had the highest ratio of nonsynonymous to synonymous substitutions (dN/dS = 1.48), followed by *SiOBP4* (dN/dS = 0.74).

**Table 1:** OBP differentiation between SB and Sb: the number of sequence‐level differences between SB and Sb and differential OBP gene expression between multiple‐ and single‐queen colonies

				Significant differential expression between colonies types	
*S. invicta* OBP locus	Nonsynonymous differences	Synonymous differences	Total differences	In queens	In workers
*SiOBP3* (*Gp‐9*)	8	1	9	Yes	No
*SiOBP4*	3	2	5	Yes	Yes
*SiOBP13*	1	1	2	Yes	Yes
*SiOBP12*	Frameshift insertion in SB and duplication in Sb			Yes	No
*SiOBPZ5*	Present exclusively in Sb				
*SiOBPZ3*	1	0	1	No	No
*SiOBP9*	0	1	1	No	No
*SiOBP16*	0	0	0	Yes	No
*SiOBP17*	0	0	0	Yes	Yes
*SiOBP15*	∼2600 bp deletion in Sb			No	No

All differentially expressed genes between social forms were overexpressed in multiple‐queen colonies.

We analyzed the OBP sequences from an outgroup species, *S. geminata*, estimated to have diverged from *S. invicta* 3–3.5 million years ago (Moreau and Bell 2013; Ward et al. 2015), that is, before the divergence between SB and Sb in *S. invicta* (estimated 0.35–0.42 million years ago; Wang et al. 2013). These sequences allowed us to determine the ancestral allele in each substitution. Sb carried the derived allele in most of the positions with nonsynonymous substitutions between SB and Sb (seven out of eight in *Gp‐9*/*SiOBP3* and all in *SiOBP4* and *SiOBPZ3*; we could not derive the two *SiOBP13* substitutions, as *S. geminata* read coverage was too low for this gene). This pattern is consistent with most nonsynonymous substitutions between SB and Sb having arisen in the lineage leading to Sb.

### COPY NUMBER AND STRUCTURAL DIFFERENTIATION BETWEEN SB AND Sb IN OBPs

We also found structural differences between SB and Sb affecting two OBPs. For the first, *SiOBP15*, we detected a ∼2600 bp deletion unique to *Sb* individuals (Fig. 2, Table 1). This deletion is derived (i.e., it is not present in the outgroup species, *S. geminata*) and causes the loss of three out of five coding exons (89 out of 139 amino acids), although it does not cause a frameshift. The second OBP with a major structural difference is *SiOBP12*. In Sb individuals, this gene is duplicated, forming the Sb‐specific *SiOBPZ5* (Fig. 2, Table 1). This gene increases the total OBP count of *S. invicta* to 24. There are 18 fixed amino acid differences between *SiOBPZ5* and the SB allele of *SiOBP12* sequence (one deleted codon, 21 nonsynonymous and four synonymous nucleotide‐level fixed differences; four codons each contain two single‐nucleotide fixed differences; dN/dS = 2.67). Intriguingly, *SiOBP12* has an early stop codon (TAG) at codon position 16 of 176 in all seven *SB* individuals and the reference genome. These individuals are also affected by six nonsynonymous SNPs and two polymorphic indels downstream of the early stop codon. *Sb* individuals have the CAG allele at position 16 of *SiOBP12*, but have a slightly later early stop codon at position 37 due to a frameshifting insertion of 17 bp at codon position 25 (nucleotide position 74). The outgroup species *S. geminata* has neither of the early stop codons. However, the very low *S. geminata* read coverage observed in the two terminal exons of this gene (median < 3; *t*_df = 999_ = −11.29, *P* < 10^−27^) could indicate a deletion in this species. *SiOBP12* is thus nonfunctional in *Sb* and *SB* individuals, and putatively nonfunctional in the outgroup species. The Sb‐specific gene *SiOBPZ5* appears to be functional as it has no early stop codons. None of the other OBPs showed differences in structure or in copy number between SB or Sb.

### FOURTEEN OBPs ARE DIFFERENTIALLY EXPRESSED BETWEEN SOCIAL FORMS

**Figure 3:**
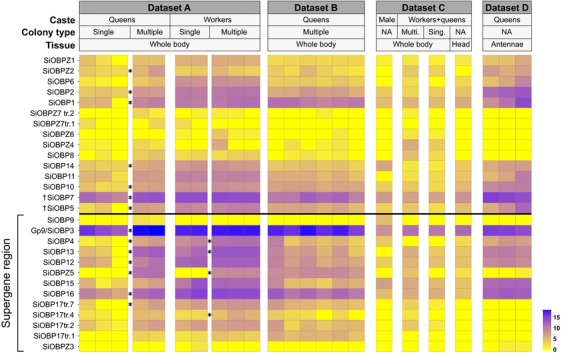
Expression patterns for all analyzed RNAseq datasets. Each tile represents the logarithm base 2 of DESeq normalized transcript counts. The rows with asterisks (*) correspond to those OBPs with significant differential expression between social forms within castes in dataset A (Morandin et al. 2016). Information about each dataset is available in Table S2 (A: PRJDB4088, B and C: PRJNA49629, D: PRJNA266847). ^†^ The exons of *SiOBP5* and *SiOBP7* are split across three unmapped scaffolds; we do not know whether these genes are within or outside the supergene region.

We compared expression levels between single‐ and multiple‐queen colonies in workers and in queens (Fig. 3; Table 1) using RNA‐Seq data from Morandin et al. (2016). General expression patterns showed an enrichment in differentially expressed genes in the supergene region in queens (expected proportion = 0.022, observed proportion = 0.059, Chi^2^_df = 1_  = 32.84, *P* = 10^−8^) but not in workers (expected proportion = 0.021, observed proportion = 0.024, Chi^2^_df = 1_ = 0.05, *P* = 0.82).

In queens, fourteen OBPs, including seven in the supergene region, were significantly differentially expressed between multiple‐queen and single‐queen colonies (DESeq2 Wald test; Benjamini–Hochberg adjusted *P* < 0.05). Consistent with this, the entire group of 24 fire ant OBPs showed significantly stronger *P*‐values for differential expression between queens from single‐ and multiple‐queen colonies than would be expected by chance (tested among 12,693 transcripts, two‐sided Kolmogorov–Smirnov test; *P* < 10^−11^; Fig. S1). Surprisingly, all of the OBPs that were differentially expressed between social forms in queens were more highly expressed in multiple‐queen colonies than in single‐queen colonies (14 significant OBPs in queens, binomial test 14 out of 14; null probability = 0.5; *P* < 10^−4^). In workers, only four OBPs (all in the supergene region) were significantly differentially expressed between social forms (DESeq2's Wald test Benjamini–Hochberg adjusted *P* < 0.05). All the differentially expressed OBPs in workers were also differentially expressed in queens. For one of these OBPs (*SiOBP17*), a different splice form was differentially expressed between colony types in queens than in workers (Fig. 3).

We additionally obtained qualitative gene expression profiles of all OBPs across 18 additional samples, in total representing seven different conditions of body part, social form, and caste (Table S2). We find generally consistent expression patterns for OBPs across all independent samples (Fig. 3). For instance, in every sample, *Gp‐9/SiOBP3* was the most highly expressed of all OBPs, whereas *SiOBPZ3* was only residually expressed (0.26 or fewer transcripts per million reads). Six OBPs had only residual expression in queen antennae and in heads, although most of these showed at least some expression in whole‐body samples. The expression of one of these genes, *SiOBP9*, appears to be limited to males.

### GENE COEXPRESSION MODULES CORRELATED WITH SOCIAL FORM

We used WGCNA (Langfelder and Horvath 2008) to produce modules of coexpressed genes from a set of worker samples (Wang et al. 2008) and a set of queen samples (Nipitwattanaphon et al. 2013). Both datasets compare *SB/SB* and *SB/Sb* samples. The queen and worker datasets, respectively, clustered into 30 and 37 coexpression modules (Table S5). Most modules in one dataset share a significant number of probes with a module in the other dataset (30 out of 31 in queens and 35 out of 37 in workers; Fisher's exact test for the overlap of the pairs of modules across the datasets, Bonferroni corrected *P* > 0.05; Fig. S2). However, in most cases there was no one‐to‐one correspondence between datasets (17 out of 31 modules in queens and 19 out of 37 in workers have significant overlaps with more than one module). Eight of the OBPs discovered in the present study are represented in the microarray (Table S5). In the worker dataset, the module “worker_D” includes four of the OBPs (*SiOBP3, SiOBP12*, *SiOBP13*, and *SiOBP16*), accounting 25% of the 16 genes in the module. OBPs were present in nine other modules, although in all nine cases the OBP represented a very small proportion of the genes in the module (Table S5).

We tested whether there were gene coexpression modules with differential eigengene expression between genotypes or social forms. In queens, four modules had differential expression between genotypes (Table S7). In workers, one module had differential eigengene expression between genotypes, and one module had differential gene expression between social forms (Table S7). One of the modules that had differential expression between genotypes in queens (“queen_X”) corresponded with the module with differential expression between genotypes in workers (“worker_Z”). Only one of the modules with differential eigengene expression includes an OBP (*SiOBP15* in “queen_D”). None of these modules were enriched for any GO term.

### THREE OBPs ARE IN A REGION OF THE GENOME WITH CHARACTERISTICS OF A RECENT SELECTIVE SWEEP

We used measurements of π among *SB* individuals in nonoverlapping 10 kb windows from Pracana et al. (2017) to determine whether any OBPs are in regions of low π, characteristic of recent selective sweeps. Among windows overlapping OBPs, two neighboring windows had π within the lower quartile of the whole‐genome distribution (π < 0.0004; Fig. S3). These two windows overlap the loci *SiOBPZ4*, *SiOBPZ7*, and *SiOBPZ6*, which are within 19 kb of each other on linkage group 3. We did not perform an equivalent analysis on *Sb* individuals because the entire region of suppressed recombination has the signature of a recent sweep in Sb (Pracana et al. 2017).

## Discussion

### THE PUTATIVE ROLE OF OBPs IN DETERMINING SOCIAL DIMORPHISM

The description of *Gp‐9* as a green beard gene (Keller and Ross 1998) and its subsequent characterization as an OBP (Krieger and Ross 2002) led to the proposal of different models of how this single gene can control the dimorphism in social organization (reviewed by Gotzek and Ross 2007). At their most basic level, these models propose that *Gp‐9* controls the production of a green‐beard odor in queens and the differential perception of this odor by workers of alternate genotypes. However, it was also proposed that *Gp‐9* additionally controls differential odor production in workers (Gotzek and Ross 2007), as well as a number of physiological and morphological traits in queens (Keller and Ross 1995; DeHeer et al. 1999; DeHeer 2002) and males (Lawson et al. 2012). The discovery that *Gp‐9* is tightly linked to hundreds of other genes (Wang et al. 2013; Pracana et al. 2017)—including the nine additional OBPs we report here—suggests that the roles previously attributed to *Gp‐9* could be split between multiple genes.

The key roles of OBPs in semiochemical perception (Leal 2013) and secretion (Li et al. 2008; Iovinella et al. 2011; Sun et al. 2012) lead to the prediction that such proteins are involved in determining the two colony types. Our results support this hypothesis, as we find divergence in protein coding sequence between SB and Sb in the OBPs in the supergene region, as well as differences in the regulation of OBP expression between single‐ and multiple‐queen colonies.

The differences in protein coding sequence affect seven of the ten OBPs in the supergene region, including *Gp‐9*/*SiOBP3*. The biggest differences are in *SiOBPZ5*, absent in SB, and in *SiOBP15*, which is missing three exons in Sb. Such differences could have a major effect on semiochemical communication. Additionally, among the four intact OBPs with nonsynonymous divergence between SB and Sb, both *Gp‐9*/*SiOBP3* and *SiOBP4* have dN/dS ratios indicative of adaptive differentiation between the alleles of these genes (Krieger and Ross 2002). This interpretation comes with some caution due to our relatively low sample size (14 individuals from an invasive population).

Additionally, 14 out of the 24 fire ant OBPs were differentially expressed between social forms in queens or in workers. Our analysis uncovers three potentially important aspects of the differential regulation of OBP expression in the two social forms. First, all of the differentially expressed OBPs are more highly expressed in multiple‐queen colonies than in single‐queen colonies, suggesting that multiple‐queen colony traits are associated with the activation of semiochemical communication pathways. Second, this activation seems to be stronger in queens, as more OBPs were differentially expressed between social forms in queens (14 OBPs) than in workers (four OBPs). This result reflects the more general pattern that the supergene region was enriched for differentially expressed genes between colony types in queens, but not in workers. The pools of workers from multiple‐queen colonies contain a mix of individuals of both genotypes (36% *SB/SB* and 64% *SB/Sb* workers expected; Buechel et al. 2014), which could mask differences between *SB/SB* workers from single‐queen colonies and *SB/Sb* workers from multiple‐queen colonies. Indeed, previous studies using cDNA microarray data and a different gene set suggest that the supergene region is enriched for differentially expressed genes in both queens (Nipitwattanaphon et al. 2013) and workers (Wang et al. 2013). Third, several of the queen‐specific differentially expressed OBPs are located outside the supergene, implying that they are regulated in *trans* by elements in the supergene. It is important to note that all three patterns could be affected by our use of samples from whole bodies, which is known to introduce several types of biases if the differences in expression are tissue specific (Johnson et al. 2013; Montgomery and Mank 2016). A particular issue is differences in allometry (i.e., relative body‐size proportions) between the individuals of different groups, for instance the larger gaster of queens in single‐queen colonies relative to queens in multiple‐queen colonies (Tschinkel 2006). These biases cannot be resolved by standard normalization methods, which are designed to normalize by entire library size rather than by the relative abundance of different transcripts (Dillies et al. 2013). Tissue‐specific gene expression profiling (Bastian et al. 2008; Robinson et al. 2013; Jasper et al. 2015) would be needed to control for such allometric differences.

Our results also support the idea that along with OBPs, other genes are likely involved in defining the social polymorphism of *S. invicta*. For instance, only one of the coexpression modules with significantly different eigengene expression contained an OBP. Furthermore, other genes inside and outside the supergene region were differentially expressed between social forms. Thus, a venue of further investigation would be to examine the potential roles of other genes, including genes from families known to be involved in communication, including chemosensory proteins (Kulmuni et al. 2013), desaturases (Helmkampf et al. 2015), fatty‐acid reductases (Lassance et al. 2010; Niehuis et al. 2013), and olfactory (Wurm et al. 2011), gustatory (Robertson et al. 2003; Zhou et al. 2012), and ionotropic receptors (Benton et al. 2009; Zhou et al. 2012). It is important to note that additional experimental work would be necessary to demonstrate whether OBPs or any of these proteins have a functional role. An interesting approach would be to measure the effect of artificially modifying the sequence or expression level of each gene to test their specific function (Gaj et al. 2013; Mohr et al. 2014).

### GENERAL EVOLUTIONARY PATTERNS OF OBPs IN *S. INVICTA*

The evolution of the OBP gene family is generally thought to follow the birth‐and‐death model, where gene duplication is followed by either the pseudogenization or the rapid functional divergence of the duplicate gene (Nei and Rooney 2005; Vieira et al. 2007). The *S. invicta* OBPs are organized in clusters along the genome, as in other insect species (Xu et al. 2003; Foret and Maleszka 2006; Vieira et al. 2007). However, none of these clusters appear to be monophyletic (Fig. 1). This is consistent with the birth–death model, where the fast evolution of genes can mask their true phylogenetic relationship (Vieira et al. 2007; Gotzek et al. 2011; Vieira and Rozas 2011). Alternative explanations include translocations affecting the OBPs during or after duplication, or ectopic gene conversion across different clusters after duplication (Arguello and Connallon 2011). Another argument in support of the birth‐and‐death model is that we find evidence of expansions in OBP number. One example is the putative ant‐specific OBP expansion reported previously (the OBP cluster including SiOBP14 in Fig. 1; Gotzek et al. 2011). We found no one‐to‐one orthologous sequences for these genes in other ants or in other arthropods (the 11 genes in this group of OBPs have BLAST similarity to only three genes in the ant *Monomorium pharaonis*; phylogenetic group 1 in Table S7). A cluster with several novel genes identified in our study (the group including *SiOBP7* and *SiOBP8* in Fig. 1) follows a similar pattern (five OBPs have BLAST similarity to one *M. pharaonis* gene, two have BLAST similarity to one *Pogonomyrmex barbatus* gene; phylogenetic group 2 in Table S7). These groups of genes may have expanded in the lineage leading to *S. invicta* and *S. geminata*, although this conclusion would require the exhaustive identification of OBPs in the present study to be replicated for other ant species. An example of a putatively recent expansion is *SiOBPZ6*, which seems to be present in multiple copies both in *S. invicta* and in *S. geminata*. Lack of heterozygosity in the region suggests that the gene copies have been recently affected by ectopic gene conversion (Arguello and Connallon 2011). Furthermore, finding that the *S. invicta SiOBPZ6* quadruplication is in a region that has a signature of a recent selective sweep makes it tempting to speculate that *SiOBPZ6* is involved in a recent adaptive process (Kondrashov 2012)—for example, to the invasive range of this species (Ascunce et al. 2011).

## Conclusion

Previous studies have focused on how the evolution of the social chromosomes has been affected by restricted recombination (Wang et al. 2013; Pracana et al. 2017), whereas the work presented here focuses on the putative mechanisms by which these chromosomes control social organization. In summary, our analyses provide a comprehensive overview of OBPs in the fire ant genome, describing patterns of differentiation and expression that are consistent with the predicted roles of OBPs in determining social organization in this species. Our study highlights the need for tissue‐specific expression profiles, as well as for broader taxonomic sampling to understand OBP evolution during the origin of the multiple‐queen colony organization. Finally, our work provides a starting point for future functional studies on the roles of OBPs in the social chromosome system.

We deposited the genomic reads of the *Solenopsis geminata* sample on NCBI SRA (SRX3045159). We manually produced gene models for 24 OBPs, which we deposited to NCBI. Additionally, all data is available at https://wurmlab.github.io/data.

Associate Editor: Z. Gompert

## Supporting information


**Figure S1**. Density distribution of the p‐values for differential expression between social forms in queens for OBPs (in green) and all other protein‐coding genes (red). The p‐values for OBPs are strongly skewed towards 0. This result is based on the expression levels from the Morandin *et al*. (2016) dataset.
**Figure S2**. Correspondence between queen and worker modules. Numbers in the table indicate probe counts in the intersection of the corresponding modules. Coloring of the table encodes − log(p), with p being the Fisher's exact test p‐value for the overlap of the two modules. A module in one dataset would be preserved across both sets if it had a single corresponding module in the other dataset with a large number of probes in common.
**Figure S3**. Nucleotide distribution (π, measured from *SB* individuals in Pracana *et al*. 2017) of 10kb windows of the assembled genome that overlap coding sequences. Vertical bars represent π of windows overlapping OBPs; orange bars representing those overlapping supergene OBPs.

**Table S1**. Summary of correspondences between identifiers of sequences produced in this project and previously published sequences, including the number of sequence differences between the two groups.
**Table S2**. Accession numbers of the gene expression data used. “Project” and “SRA” columns indicate NCBI identifiers. The descriptions provided and the sequencing method used are based on metadata available on NCBI and in the manuscripts. Two samples (marked with an asterisk) were discarded because of very low coverage after aligning the reads to the S. invicta genome.
**Table S3**. Table showing the results of the alignment of ESTs from the *S.invicta* microarray (Platform GPL6930) to its reference genome. The columns “Scaffold”, “Start” and “End” indicate the coordinates of the EST in the reference. “Probe” refers to probe to which each EST belongs and “Gene” refers to the gene intersecting that particular region of the *S.invicta* genome. ESTs tagged with an asterisk (*) map to more than one location in the genome.
**Table S4**. Details of closest BLASTP hit to NCBI “nr” database for each newly produced *S. invicta* OBP sequence.
**Table S5**. Number of genes represented in each co‐expression module; OBPs represented in each module. Each gene may be represented by multiple probes. Probes in queen_E_1 are not assinged to a module.
**Table S6**. Gene co‐expression modules with module eigene differential expression between genotypes in queens (*SB/SB* versus *SB/Sb*), between genotypes in workers from multiple‐queen colonies (*SB/SB* versus *SB/Sb*), and betwen social forms in *SB/SB* workers (single‐queen colony versus multiple‐queen colony). Differential expression was tested with t‐tests within each comparison within each dataset, with p‐values corrected for multiple testing using Bonferroni correction.
**Table S7**. Putative OBP orthologs in other species. First, we ran a tblastn search of all *S. invicta* OBPs against all non‐*S. invicta* arthropod sequences, accepting hits where e‐value < 10^−3^. We then ran a blastx search of these hits against the *S. invicta* gene predictions (including our newly curated OBP set). We report the hits with the lowest e‐value of the blastx search. We repeated this analysis by searching non‐ant arthropods (not Formicidae). The coloured cells represent cases where the same non‐*S. invicta* sequence aligns to multiple *S. Invicta* OBPs.
