# Supplementary material for: Fire ant social chromosomes: Differences in number, sequence and expression of odorant binding proteins
Source: Evol Lett. 2017 Aug 23;1(4):199–210. doi: 10.1002/evl3.22 (PMC6121795; doi:10.1002/evl3.22)
Supplement: Supplementary file 1 — Figure S1. Density distribution of the p‐values for differential expression between social forms in queens for OBPs (in green) and all other protein‐coding genes (red). The p‐values for OBPs are strongly skewed towards 0. This result is based on the expression levels from the Morandin et al. (2016) dataset. Figure S2. Correspondence between queen and worker modules. Numbers in the table indicate probe counts in the intersection of the corresponding modules. Coloring of the table encodes − log(p), with p being the Fisher's exact test p‐value for the overlap of the two modules. A module in one dataset would be preserved across both sets if it had a single corresponding module in the other dataset with a large number of probes in common. Figure S3. Nucleotide distribution (π, measured from SB individuals in Pracana et al. 2017) of 10kb windows of the assembled genome that overlap coding sequences. Vertical bars represent π of windows overlapping OBPs; orange bars representing those overlapping supergene OBPs. [file EVL3-1-199-s001.pdf]

Fire Ant Social Chromosomes: Differences in Number, Sequence and Expression of Odorant Binding Proteins

Supplementary Material

Supplementary Methods and Supplementary Figures

Authors: Rodrigo Pracana*, Ilya Levantis*, Carlos Martínez-Ruiz, Eckart Stolle, Anurag Priyam, Yannick Wurm

* Joint first authors

Affiliation: School of Biological and Chemical Sciences, Queen Mary University of London, London, UK

Contact information for corresponding authors:

Yannick Wurm

School of Biological and Chemical Sciences,
Queen Mary University of London,
Mile End Road, E1 4NS, London,
United Kingdom.
Tel: +44 75145 33020
Skype: yannickwurm
Email: y.wurm@qmul.ac.uk

Rodrigo Pracana

School of Biological and Chemical Sciences,
Queen Mary University of London,
Mile End Road, E1 4NS, London,
United Kingdom.
Tel: +44 70236 66332

Email: r.pracana@qmul.ac.uk

Supplementary Methods

OBP discovery and manual gene model curation

We used MAKER2 (v2.31; Cantarel *et al.* 2008) to generate consensus gene models for the *S. invicta* genome assembly (Wurm *et al.* 2011) from TopHat2 (v2.0.11; Kim *et al.* 2013) alignments of RNASeq reads (SRA accessions SRX757226-SRX757228) to the reference genome, an assembly of fire ant Expressed Sequence Tag (EST) libraries, protein sequences from SwissProt (downloaded June, 2014), *A. mellifera* (amel_OGSv3.2_pep.fa) and *N. vitripennis* (Nvit_OGSv1.2_pep.fa) genome projects, and *de novo* predictions from SNAP (Korf 2004) and Augustus (Stanke *et al.* 2006) using HMM models that had been generated during the fire ant genome project (Wurm *et al.* 2011). To identify regions of the genome putatively containing OBPs, we performed blastn and tblastn (Camacho *et al.* 2009; Priyam *et al.* 2015) searches of the fire ant genome on antgenomes.org (Wurm *et al.* 2009) using as queries previously published fire ant OBP sequences (Gotzek *et al.* 2011) and Uniprot sequences that are part of the Pfam family 'PBP_GOBP' (Finn *et al.* 2014; UniProt Consortium 2015). We integrated all aforementioned data using the genomic annotation editor Afra (Priyam unpublished), the genome browser JBrowse (Skinner *et al.* 2009), the tool GeneValidator (which assesses the quality of annotations by comparing them to public databases; Drăgan *et al.* 2016) and custom scripts. Manual curation followed a the standard approach based on Web Apollo (Lee *et al.* 2013), including the inspection and adjustment of exon boundaries to ensure that the exon-intron structure of gene models was consistent with mappings of RNA sequence reads, and that the gene models had canonical splice sites, translation start and stop sites, and appropriate open reading frames. We also identified alternative spliced transcripts by visualising the alignments of the RNA sequence reads. After producing high quality gene models through this method, we used these sequences for further blastn and tblastn searches against the reference genome to identify further putative OBPs. These were curated as above; this process was repeated iteratively until no new putative OBP loci were discovered.

Our pipeline identified seventeen out of the eighteen OBP genes that had been previously reported, although eight had differences in sequence and/or length relative to the published sequences (Table S1). We found no genomic region with more than 80% identity to the remainder gene, *SiOBP18* (Table S1), which had been identified from a single Sanger-sequenced EST (Wang et al. 2007; Gotzek et al. 2011), suggesting that this gene is either missing from the reference genome assembly, or that the sequence of the original EST was an artefact. We found evidence of alternative splicing for *SiOBP17* (four splice forms) and for the newly discovered *SiOBPZ7* (two splice forms). We found no support for the suggestion that *SiOBP12* and *SiOBP13* share an exon (Zhang *et al.* 2016). All the genomic and transcriptomic sequences analysed in the OBP discovery pipeline included an insertion relative to the reference assembly affecting *SiOBP14* (insertion of a T in position NW_011802221.1:1,287,729), suggesting an error in the assembly. The sequence reported for this gene includes this insertion.

We used a genetic map (Pracana *et al.* 2017) to assign OBPs to linkage groups. We were able to position 20 of the OBPs in linkage groups, including all novel OBPs

(Fig. 1). Four OBPs were in unmapped scaffolds. Of these, *SiOBP9* is in a scaffold that we previously classed as putatively in the supergene (in Pracana et al. 2017) given its high SB-Sb differentiation. *SiOBP2* was in a scaffold without any divergence, thus classified as outside the supergene. It was not possible to confidently determine the positions of the remaining two OBPs (*SiOBP5* and *SiOBP7*), as each had exons in multiple small unmapped scaffolds.

Phylogenetic analysis

S. invicta OBPs are a highly divergent gene family (Gotzek et al. 2011). We aligned the coding sequences of the 24 *S. invicta* OBPs using MAFFT-linsi (v6.903b; Katoh & Toh 2008). We removed ambiguous sections from this alignment using trimAL (v1.4.1; Capella-Gutiérrez et al. 2009) with the -gappyout option and built a "guide" tree using RaxML (v8.2.9; Stamatakis 2006) with the GTRGAMMAI model. We then used PRANK (v120626; Löytynoja & Goldman 2005) to generate a codon-level alignment of the original sequences, guided by the tree obtained above. Using the same parameters as above, we removed ambiguous sections from this alignment using trimAl and built a final tree using RaXML (10,000 bootstraps).

Read filtering of *S. invicta* whole-genome sequences

We used whole-genome sequences from one *SB* and one *Sb* male from each of seven colonies that had been sequenced at low coverage (Illumina 2*100bp paired-end genome shotgun sequences; ~6x-8x coverage) in 2012 (NCBI SRP017317) (Wang *et al.* 2013). Each of these samples is a haploid male (ants have a haplo-diploid sex determination system), and the sequencing coverage is sufficiently homogeneous (Pracana *et al.* 2017) for the analysis reported here, including high confidence genotype calling. We used seqtk (v1.0-r31; https://github.com/lh3/seqtk) to trim 2bp from the start and 5bp from the ends of the reads. We removed any read where more than 25% of the bases had a quality score smaller than 25 using fastq_quality_filter in the fastx toolkit (0.0.14; http://hannonlab.cshl.edu/fastx_toolkit). We used GNU parallel to parallelise this pipeline (Tange 2011).

Detection of copy number and structural variation in OBPs

We used bowtie2 (v2.1.0; Langmead & Salzberg 2012) to align the cleaned reads to the reference genome assembly. We visually inspected the alignments of each of our curated gene predictions, searching for regions with no coverage to identify deletions and high coverage to identify duplications.

The genomic region that includes three exons of *SiOBP15* (scaffold NW_011801067.1:293,460-296,015) had no reads in any *Sb* individual, consistent with a deletion of this region (it is impossible to determine the exact size of the deletion as the region is directly upstream of a non-assembled portion of the scaffold). We observed no other such pattern of deletion.

SiOBP12 (which is within the supergene region) had approximately two times higher coverage in *Sb* individuals relative to *SB* individuals. Approximately half of the reads from *Sb* individuals had a small number of consistent sequence differences to the other

reads. This suggested that a recent duplication of the gene occurred. To obtain consensus sequences for *Sb* individuals for both duplicates, we extracted all pairs of reads from *Sb* individuals for which at least one pair mapped to the contig containing the transcribed region of *SiOBP12* and performed *de novo* assembly using MIRA (v4.0.2; Chevreux *et al.* 1999). This resulted in assemblies on separate contigs of two genes: *SiOBP12* and the Sb-specific duplicate we named *SiOBPZ5*.

Visual inspection of *SiOBPZ6* (outside the supergene region) revealed that this gene had a much higher number of mapped reads than other genes. To estimate the number of copies of *SiOBPZ6*, we measured the median coverage per base pair of the seven *SB* individuals for this gene and for 1000 additional randomly sampled genes using bedtools coverage (with argument -d; v2.25.0; Quinlan & Hall 2010). For each individual, we calculated the ratio between the coverage of *SiOBPZ6* and the mean coverage of the 1000 random genes. For a single-copy gene, we expect these ratios to be one; we used a one-sample t-test to determine if the distribution of these ratios had a mean different from one. We did not produce individual sequences for each *SiOBPZ6* copy because there was an insufficient number of variable sites to differentiate the copies. The sample used for genome assembly (NCBI SAMN00014755; Wurm *et al.* 2011) was not included in this test because it was sequenced using an earlier (noisier) Illumina technology.

Orthology in other species

Using a reciprocal blast approach, we searched for the closest orthologous sequence of each OBP gene. First, we ran a tblastn search of all *S. invicta* OBPs against all non-*S. invicta* arthropod sequences available on NCBI nr on 2017-03-21, accepting hits where e-value < 10^{-3}. We then ran a blastx search of these hits against the *S. invicta* gene predictions (including our newly curated OBP set). We report the hits with the lowest e-value (Table S7). We repeated this analysis by searching non-ant arthropods (not Formicidae).

Variant Calling in *S. invicta* OBPs

We added the contig with the *Sb*-specific *SiOBPZ5* to the reference assembly. Using bowtie2 (v2.1.0; Langmead & Salzberg 2012), we aligned the cleaned reads of the seven *Sb* individuals (see above) to the revised assembly and the seven *SB* individuals to the original reference assembly. We called single nucleotide polymorphisms (SNPs) in the protein coding regions of the supergene OBPs using samtools mpileup (Li *et al.* 2009) and bcftools call (with arguments --ploidy 1 and –m; v1.3.1; https://samtools.github.io/bcftools/bcftools.html). We manually inspected the read alignments at each SNP position using the genome viewer IGV (Thorvaldsdóttir *et al.* 2013).

Sequencing and variant calling of the OBPs of an outgroup species

We produced whole-genome sequencing reads of the outgroup species *Solenopsis geminata*. DNA was extracted from a pool of ten workers (sampled in Thailand by Dr Adam Devenish, University College London, United Kingdom) using the Phenol-Chloroform method in Hunt and Page (1994) and sequenced using Illumina

HiSeq 4000 (x11 coverage). We filtered the reads using skewer (v0.2.2; Jiang *et al.* 2014), with the following parameters: --mean-quality 20, --end-quality 15, -l 100, -n yes and -r 0.1. The reads were aligned to the *S. invicta* reference genome assembly using bowtie2 (v2.1.0; Langmead & Salzberg 2012). All OBPs were covered by *S. geminata* reads, although there was very low coverage (median coverage < 3) in the two terminal exons of *SiOBP12* and *SiOBP13*. Freebayes (v1.0.2-33-gdbb6160; Garrison & Marth 2012) was used to call variants between the sample and the reference assembly in the regions within 1000 bp of each OBP (excluding the two terminal exons of *SiOBP12* and *SiOBP13*). We filtered the variants using the parameter RO < 2, chosen based on visual inspection of the alignment using IGV (Thorvaldsdóttir *et al.* 2013), and limited our analysis to homozygous positions within the coding sequence of each OBP.

Gene expression of *S. invicta* OBPs in publically available RNA sequencing datasets

We analysed all available RNA sequencing (RNA-Seq) data from the NCBI SRA database for *S. invicta* as of January 2017 (data from Wurm *et al.* 2011; Morandin *et al.* 2016 and PRJNA266847; details in Table S2). These included Illumina and Roche 454 sequences. Read quality was assessed using fastQC (v0.11.5; http://www.bioinformatics.babraham.ac.uk/projects/fastqc/). Low quality bases were removed using the default options in fqtrim (v0.9.5; http://ccb.jhu.edu/software/fqtrim/).

We determined the expression levels of *S. invicta* transcripts using count mode in Kallisto (v0.43.0; Bray *et al.* 2016). As a reference, we modified the *S. invicta* protein-coding gene annotation release 100 (NCBI) by removing all automatically annotated OBPs and instead adding the OBP sequences we manually curated above. We masked regions of *SiOBP12* and *SiOBPZ5* that were identical between these recent duplicates to prevent reads from one gene to be misassigned to the other. *SiOBP15* lacks three exons in its Sb variant, so to prevent misalignment, we treated each variant of *SiOBP15* as a different transcript. The total read count for *SiOBP15* is the sum of its two variants. To control for the potential effects of sequence differences between SB and Sb, we repeated the analysis twice: first using the SB alleles of the OBPs, then using the Sb alleles. We only show the analysis done using the Sb alleles of the OBPs because both analyses produced qualitatively identical results.

For paired-end reads, we used the default counting options of Kallisto. For single-end reads, we provided Kallisto the average fragment length of each sample (as indicated on NCBI SRA) and we set the estimated standard deviation to 20 bp. To be able to analyse at least >50% of low-coverage Roche 454 reads with Kallisto, we set the average and the standard deviation of fragment length to 1.

We used Tximport (v1.2.0; Soneson *et al.* 2015) to import the estimated counts produced by Kallisto into the R (R Core Team 2016) implementation of DESeq2 (v1.14.1; Love *et al.* 2014). Each sample was independently normalised using the DESeq2 method. Additionally, we performed genome-wide analysis of differential expression on data from (Morandin *et al.* 2016) using a standard DESeq2 approach to identify expression differences between social forms in queens and in workers. Queens and workers were analysed separately because they were sampled using different collection methods, which resulted in different variance patterns in each dataset (Morandin et al. 2016). We included the Sb-specific *SiOBPZ5* in the analysis as a

control. As expected, this gene is significantly differentially expressed between single- and multiple-queen colonies in both workers and queens. We performed a standard Chi^2 test to determine whether the supergene region is enriched in differentially expressed loci relative to the rest of the genome.

Differential expression of gene co-expression modules across social forms

We created gene co-expression modules from two microarray sets comparing single-queen with multiple-queen colonies, one with queen samples (GSE42062; Nipitwattanaphon *et al.* 2013) and the other with worker samples (E-GEOD-11694; Wang *et al.* 2008). We did not use the RNAseq data because it does not include a sufficient number of samples of each social form to create gene co-expression modules. Both microarray sets use the same microarray platform (Platform GPL6930), which includes 25,344 probes (Wang *et al.* 2007). To determine the number of genes that these probes represented, we aligned the sequences of all probes against the gnG assembly for *S. invicta* using the 'est2genome' mode with a minimum 95% identity in Exonerate (v2.2.0; Slater & Birney 2005). The positions of the probes in the genome were then intersected against the annotation release 100 for *S. invicta* used in the rest of analyses with the R package 'GenomicRanges' (Lawrence *et al.* 2013). The probe sequences intersected with 3,673 unique genes.

We downloaded the normalised expression values of each dataset from NCBI GEO. For the queen set, we removed the 16 samples with reproductive age class because *SB/SB* reproductive samples had very low variance in gene expression relative to individuals of other age classes. The remaining set included 31 *SB/SB* samples and 31 *SB/Sb* samples (all virgin queens originating from multiple-queen colonies). The worker set included 20 samples from single-queen colonies and 40 from multiple-queen colonies (20 *SB/SB* and 20 *SB/Sb*; we removed two *Sb/Sb* samples). For each set, we removed any probe that had "null" expression in more than five individuals. For the remaining probes, individuals with "null" expression were imputed to the median expression of the probe. After filtering, there were 18,291 probes in common between the two datasets, representing 3,046 genes. We used the ComBat function in the sva R library (v3.18.0; Leek *et al.* 2012) to adjust both sets for the year in which the microarrays were produced. We used Weighted Gene Co-expression Network Analysis (v1.49; Langfelder & Horvath 2008) to create signed modules for each set. We used a soft-thresholding power of 5 for both sets. Modules were detected using the Dynamic Tree Cut method and merged using an eigengene dissimilarity threshold of 0.3. We used t-tests to determine whether any module eigengene is correlated with genotype or social form. In queens, we compared *SB/SB* to *SB/Sb* samples because all originate in multiple-queen colonies. In workers, we separated the effect of genotype from the effect of social form following the approach in Wang *et al.* (Wang *et al.* 2008): we compared genotypes (*SB/SB* versus *SB/Sb*) using samples from multiple-queen colonies, and we compared across social forms (single-queen versus multiple-queen) using *SB/SB* samples only, and corrected with the p-values Bonferroni correction. In the worker dataset, *SB/Sb* samples originate from both single- and multiple-queen colonies, so we also tested whether any module eigengene is correlated with social form in this dataset.

Gene Ontology (GO) term annotation of the *Solenopsis invicta* genome

We used the modified *S. invicta* annotations created for the RNAseq alignments (above) as a query for blastp against the nr database of NCBI. We limited the hits to the 20 best matches, with a minimum e-value of 10^{-5}. The results were used with Blast2GO (v4.1.9; Conesa *et al.* 2005) to obtain the GO terms for each protein coding gene in *S.invicta*. We tested whether any GO terms were overrepresented in any co-expression modules that were significantly correlated with social form or genotype using TopGO (v2.26.0; Alexa & Rahnenfuhrer 2016) with the 'elim' algorithm and a Fisher's exact test, with p-values corrected for multiple-testing (Benjamini & Hochberg 1995).

Evidence for selection based on nucleotide diversity

Genomic regions that underwent recent selective sweeps are characterised by low nucleotide diversity (π) (Smith & Haigh 1974; Nei 1987; Nachman 2001). We used measurements of π along a sliding window of the genome, originally produced by Pracana et al. (2017), to identify selection pressure acting on S. invicta OBPs. These measurements were produced from SNPs identified de novo from the 7 SB samples mentioned above and an additional SB sample (NCBI SAMN00014755, ~33x coverage) using Cortex (v1.0.5.20; Iqbal et al. 2012). Measurements of π were taken from non-overlapping 10kb windows. *Sb* samples were excluded to avoid measuring diversity across sibling pairs, and because of the very low diversity in the Sb supergene variant ($\pi \approx 0$), which may be the result of low recombination in Sb and a putative recent fixation of this variant in the sampled population (Pracana et al. 2017).

References

1.
Bray, N.L., Pimentel, H., Melsted, P. & Pachter, L. (2016). Near-optimal probabilistic RNA-seq quantification. *Nat. Biotechnol.*, 34, 525–527.

2.
Camacho, C., Coulouris, G., Avagyan, V., Ma, N., Papadopoulos, J., Bealer, K., *et al.* (2009). BLAST+: architecture and applications. *BMC Bioinformatics*, 10, 421.

3.
Cantarel, B.L., Korf, I., Robb, S.M.C., Parra, G., Ross, E., Moore, B., *et al.* (2008). MAKER: an easy-to-use annotation pipeline designed for emerging model organism genomes. *Genome Res.*, 18, 188–196.

4.
Chevreux, B., Wetter, T. & Suhai, S. (1999). Genome sequence assembly using trace signals and additional sequence information. In: *Computer Science and Biology: Proceedings of the German Conference on Bioinformatics GCB'99*. Heidelberg, pp. 45–56.

5.
Conesa, A., Götz, S., García-Gómez, J.M., Terol, J., Talón, M. & Robles, M. (2005). Blast2GO: a universal tool for annotation, visualization and analysis in functional genomics research. *Bioinformatics*, 21, 3674–3676.

6.
Drăgan, M.-A., Moghul, I., Priyam, A., Bustos, C. & Wurm, Y. (2016). GeneValidator: identify problems with protein-coding gene predictions. *Bioinformatics*, 32, 1559–1561.

7.
Finn, R.D., Bateman, A., Clements, J., Coggill, P., Eberhardt, R.Y., Eddy, S.R., *et al.* (2014). Pfam: the protein families database. *Nucleic Acids Res.*, 42, D222–30.

8.
Garrison, E. & Marth, G. (2012). Haplotype-based variant detection from short-read sequencing. *arXiv [q-bio.GN]*.

9.
Gotzek, D., Robertson, H.M., Wurm, Y. & Shoemaker, D. (2011). Odorant binding proteins of the red imported fire ant, *Solenopsis invicta*: an example of the problems facing the analysis of widely divergent proteins. *PLoS One*, 6, e16289.

10.
Jiang, H., Lei, R., Ding, S.-W. & Zhu, S. (2014). Skewer: a fast and accurate adapter trimmer for next-generation sequencing paired-end reads. *BMC Bioinformatics*, 15, 182.

11.
Kim, D., Pertea, G., Trapnell, C., Pimentel, H., Kelley, R. & Salzberg, S.L. (2013). TopHat2: accurate alignment of transcriptomes in the presence of insertions, deletions and gene fusions. *Genome Biol.*, 14, R36.

12.
Korf, I. (2004). Gene finding in novel genomes. *BMC Bioinformatics*, 5, 59.

13.
Langfelder, P. & Horvath, S. (2008). WGCNA: an R package for weighted correlation network analysis. *BMC Bioinformatics*, 9, 559.

14.
Langmead, B. & Salzberg, S.L. (2012). Fast gapped-read alignment with Bowtie 2. *Nat. Methods*, 9, 357–359.

15.
Lawrence, M., Huber, W., Pagès, H., Aboyoun, P., Carlson, M., Gentleman, R., *et al.* (2013). Software for computing and annotating genomic ranges. *PLoS Comput. Biol.*, 9, e1003118.

16.
Lee, E., Helt, G.A., Reese, J.T., Munoz-Torres, M.C., Childers, C.P., Buels, R.M., *et al.* (2013). Web Apollo: a web-based genomic annotation editing platform. *Genome Biol.*, 14, R93.

17.
Leek, J.T., Johnson, W.E., Parker, H.S., Jaffe, A.E. & Storey, J.D. (2012). The sva package for removing batch effects and other unwanted variation in high-throughput experiments. *Bioinformatics*, 28, 882–883.

18.
Li, H., Handsaker, B., Wysoker, A., Fennell, T., Ruan, J., Homer, N., *et al.* (2009). The

Sequence Alignment/Map format and SAMtools. *Bioinformatics*, 25, 2078–2079.

19.
Love, M.I., Wolfgang, H. & Simon, A. (2014). Moderated estimation of fold change and dispersion for RNA-seq data with DESeq2. *Genome Biol.*, 15, 550.

20.
Morandin, C., Tin, M.M.Y., Abril, S., Gómez, C., Pontieri, L., Schiøtt, M., *et al.* (2016). Comparative transcriptomics reveals the conserved building blocks involved in parallel evolution of diverse phenotypic traits in ants. *Genome Biol.*, 17, 43.

21.
Nipitwattanaphon, M., Wang, J., Dijkstra, M.B. & Keller, L. (2013). A simple genetic basis for complex social behaviour mediates widespread gene expression differences. *Mol. Ecol.*, 22, 3797–3813.

22.
Pracana, R., Priyam, A., Levantis, I., Nichols, R. & Wurm, Y. (2017). The fire ant social chromosome supergene variant Sb shows low diversity but high divergence from SB. *Molecular Ecology*, doi:10.1111/mec.14054.

23.
Priyam, A. (unpublished). Afra.

24.
Priyam, A., Woodcroft, B.J., Rai, V., Munagala, A., Moghul, I., Ter, F., *et al.* (2015). Sequenceserver: a modern graphical user interface for custom BLAST databases. *bioRxiv*, doi:10.1101/033142.

25.
Quinlan, A.R. & Hall, I.M. (2010). BEDTools: a flexible suite of utilities for comparing genomic features. *Bioinformatics*, 26, 841–842.

26.
R Core Team. (2016). *R: A Language and Environment for Statistical Computing*. R Foundation for Statistical Computing.

27.
Skinner, M.E., Uzilov, A.V., Stein, L.D., Mungall, C.J. & Holmes, I.H. (2009). JBrowse: a next-generation genome browser. *Genome Res.*, 19, 1630–1638.

28.
Slater, G.S.C. & Birney, E. (2005). Automated generation of heuristics for biological sequence comparison. *BMC Bioinformatics*, 6, 31.

29.
Soneson, C., Love, M.I. & Robinson, M.D. (2015). Differential analyses for RNA-seq: transcript-level estimates improve gene-level inferences. *F1000Res.*, 4, 1521.

30.
Stanke, M., Schöffmann, O., Morgenstern, B. & Waack, S. (2006). Gene prediction in eukaryotes with a generalized hidden Markov model that uses hints from external sources. *BMC Bioinformatics*, 7, 62.

31.
Tange, O. (2011). GNU parallel - the command-line power tool. *The USENIX Magazine*, 36, 42–47.

32.
Thorvaldsdóttir, H., Robinson, J.T. & Mesirov, J.P. (2013). Integrative Genomics Viewer (IGV): high-performance genomics data visualization and exploration. *Brief. Bioinform.*, 14, 178–192.

33.
UniProt Consortium. (2015). UniProt: a hub for protein information. *Nucleic Acids Res.*, 43, D204–212.

34.
Wang, J., Jemielity, S., Uva, P., Wurm, Y., Gräff, J. & Keller, L. (2007). An annotated cDNA library and microarray for large-scale gene-expression studies in the ant *Solenopsis invicta*. *Genome Biol.*, 8, R9.

35.
Wang, J., Ross, K.G. & Keller, L. (2008). Genome-wide expression patterns and the genetic architecture of a fundamental social trait. *PLoS Genet.*, 4, e1000127.

36.
Wang, J., Wurm, Y., Nipitwattanaphon, M., Riba-Grognuz, O., Huang, Y.C., Shoemaker, D., *et al.* (2013). A Y-like social chromosome causes alternative colony organization in fire ants. *Nature*, 493, 664–668.

37.
Wurm, Y., Uva, P., Ricci, F., Wang, J., Jemielity, S., Iseli, C., *et al.* (2009). Fourmidable: a database for ant genomics. *BMC Genomics*, 10, 5.

38.
Wurm, Y., Wang, J., Riba-Grognuz, O., Corona, M., Nygaard, S., Hunt, B.G., *et al.* (2011). The genome of the fire ant Solenopsis invicta. *Proc. Natl. Acad. Sci. U. S. A.*, 108, 5679–5684.

39.
Zhang, W., Wanchoo, A., Ortiz-Urquiza, A., Xia, Y. & Keyhani, N.O. (2016). Tissue, developmental, and caste-specific expression of odorant binding proteins in a eusocial insect, the red imported fire ant, *Solenopsis invicta*. *Sci. Rep.*, 6, 35452.

Supplementary Figures

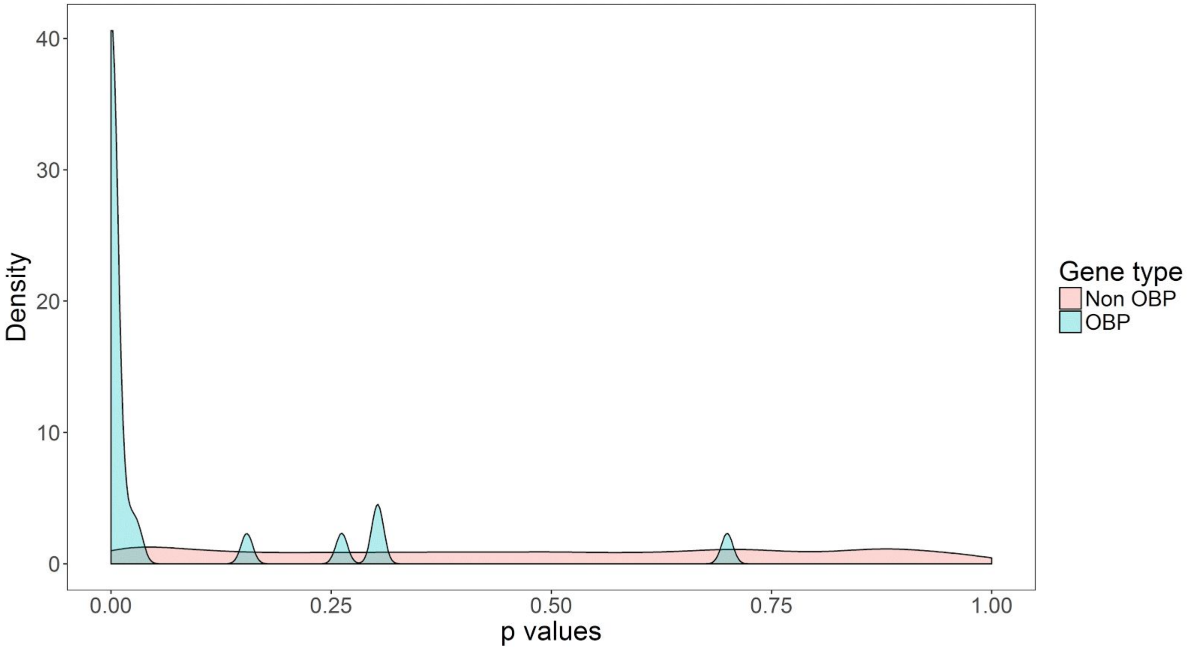

Figure S1: Density distribution of the p-values for differential expression between social forms in queens for OBPs (in green) and all other protein-coding genes (red). The p-values for OBPs are strongly skewed towards 0. This result is based on the expression levels from the Morandin *et al.* (2016) dataset.

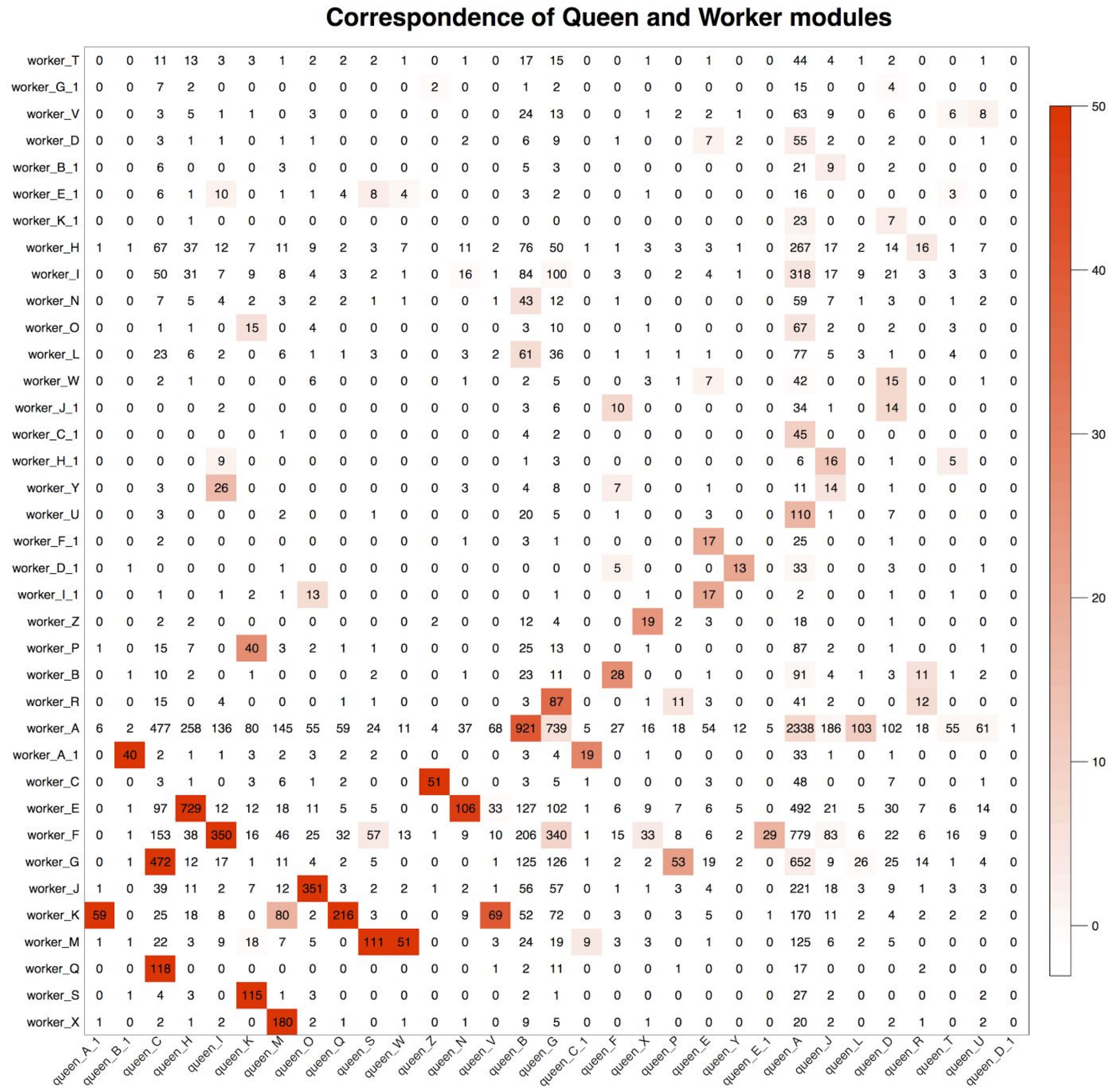

Figure S2: Correspondence between queen and worker modules. Numbers in the table indicate probe counts in the intersection of the corresponding modules. Coloring of the table encodes − log(p), with p being the Fisher's exact test p-value for the overlap of the two modules. A module in one dataset would be preserved across both sets if it had a single corresponding module in the other dataset with a large number of probes in common.

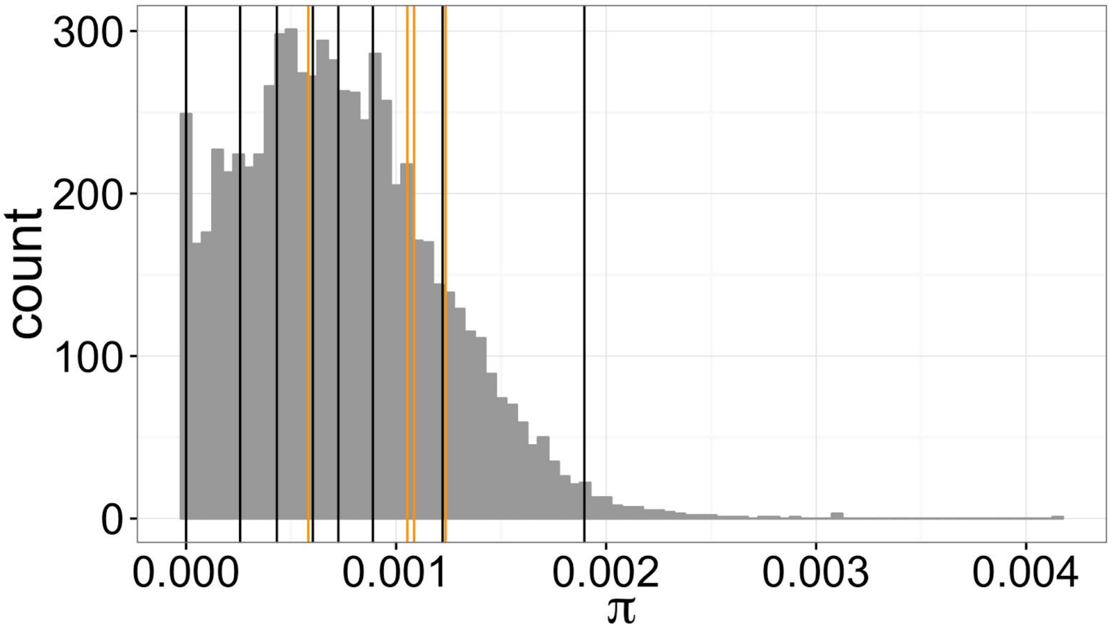

Figure S3: Nucleotide distribution (π, measured from *SB* individuals in Pracana *et al.* 2017) of 10kb windows of the assembled genome that overlap coding sequences. Vertical bars represent π of windows overlapping OBPs; orange bars representing those overlapping supergene OBPs.
